# Injuries Caused by Explosion of Electronic Cigarette Devices

**Published:** 2016-02-29

**Authors:** C. Alessandra Colaianni, Luis F. Tapias, Ryan Cauley, Robert Sheridan, John T. Schulz, Jeremy Goverman

**Affiliations:** Division of Burn Surgery, Department of Surgery, Massachusetts General Hospital, Boston

**Keywords:** e-cigarette, burn, skin graft, trauma, public health

## DESCRIPTION

Three men (aged 41, 26, and 18 years) presented with traumatic injuries caused by spontaneous explosion of electronic cigarette devices. Two individuals required skin grafting for third-degree burns after the devices exploded in their pockets; the other sustained facial lacerations and tooth fractures after the device exploded in his mouth.

## QUESTIONS

1. **What are e-cigarettes?**
2. **What is known about the health risks associated with e-cigarette use?**
3. **What traumatic injury patterns are associated with e-cigarette use?**
4. **Are e-cigarette users aware of the risks of spontaneous explosion?**

## DISCUSSION

Electronic cigarettes, or e-cigarettes, convert a nicotine-containing liquid solution (e-liquid) into a vapor without burning, instead using a battery-powered heating element.^1^^,^^2^ They are designed to resemble traditional cigarettes both in design and in operation, but are thought to be healthier than cigarettes due to the lack of combustion and/or carcinogenic additives; indeed, the majority of end-users view e-cigarettes as a smoking cessation aid.^3^^,^^4^ Awareness and use of e-cigarettes have increased significantly over the past 10 years as a result of sustained marketing campaigns touting the possible health benefits of smokers replacing cigarettes with e-cigarettes.^4^

Numerous studies, however, have noted that very little is actually known about the long-term health consequences of e-cigarette use.^5^ Furthermore, e-cigarettes vary widely in terms of product design and are currently largely unregulated.^1^ While e-cigarettes have been linked in some preliminary studies to decreased use of traditional cigarettes, the devices themselves may pose unrecognized risks to end-users that are currently poorly understood.^6^ For example, many types of e-cigarettes use lithium batteries because they are compact and potent—but as one study noted, lithium batteries are inherently susceptible to a condition called “thermal runaway,” which occurs when the internal battery temperature increases to the point of causing an internal fire or explosion.^1^ When combined with the inherent flammability of e-liquid, this could represent a major public health concern.

Over the time span of 2 months, our hospital treated 3 separate individuals who presented with traumatic injuries from e-cigarettes that were directly associated with lithium battery explosion. Injury patterns that we treated included thermal injuries and blast injuries. Locations of injury included extremities (from storing the device in the pockets) and oral/maxillofacial (from device exploding during use). Two patients were carrying the device in their pockets when they spontaneously exploded, causing third-degree burn injuries to their legs and second-degree burn injuries to their genitalia and/or hands. Given the blast component to this injury, and the questionable viability of the wound bed, these 2 patients were initially excised and temporarily covered with cadaveric allograft. They returned to the operating room approximately 5 days later for removal of allograft, further excisional debridement, and placement of autograft. The third individual was using the device when it exploded. He was initially intubated for airway protection and suffered traumatic avulsion and fracture of several teeth and lacerations of the tongue and upper lip, requiring suture repair.

[**Click Here to view video**]

From 2009 to 2014, the Food and Drug Administration has received 4 reports of e-cigarette explosion, 3 of which included significant thermal injury and/or blast injury, as well as damage to personal property caused when the devices exploded while charging in users' homes or cars.^7^ There are no current data or consistent reporting mechanism for e-cigarette explosion, so it is impossible to know how many incidents occur annually. Although e-cigarette devices are not currently well-regulated, one study looked at current practice in package labeling with regard to product use and disposal.^2^ The authors examined packaging of 9 brands of e-cigarettes. Batteries were only mentioned with regard to proper disposal, not with regard to risk of explosion. Therefore, one may infer that e-cigarette users are not well informed of the risk from explosion of lithium batteries.

In conclusion, while e-cigarettes are in widespread use and are largely viewed as safe and healthy alternatives to traditional cigarettes, the devices themselves are unregulated and carry unrecognized safety risks. Lithium batteries, which are used in many e-cigarette devices, carry inherent risk of flame and explosion that are currently not well marked on product packaging and which can cause significant morbidity among end-users in the form of thermal and blast injuries as well as damage to personal property. Further regulation is necessary to educate end-users on the potential risks of e-cigarette use as well as to accurately track the incidence of injuries.

## Figures and Tables

**Figure 1:**
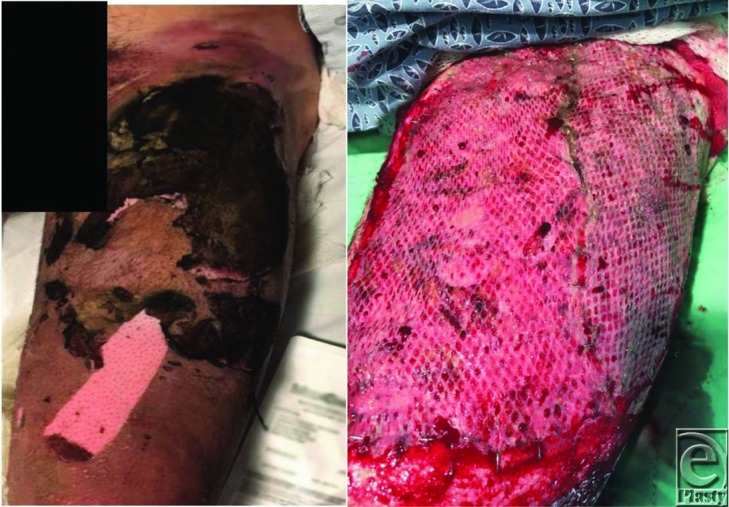
Left thigh on admission (left) and postoperative day 5 after excision and autografting (right).

**Figure 2:**
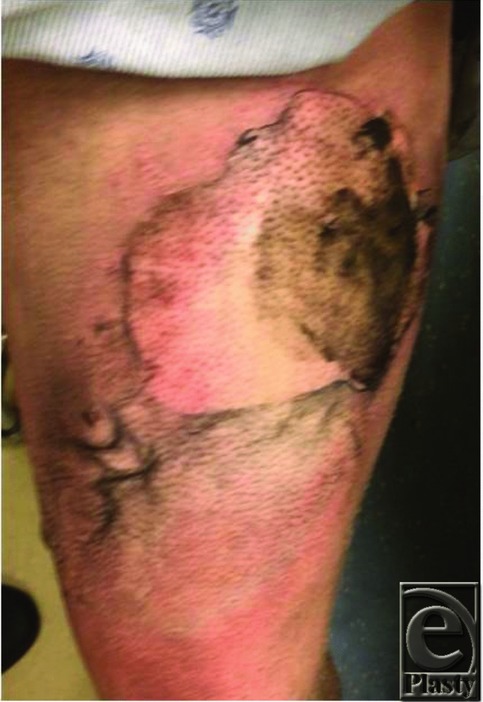
Left thigh admission photograph.

**Figure 3:**
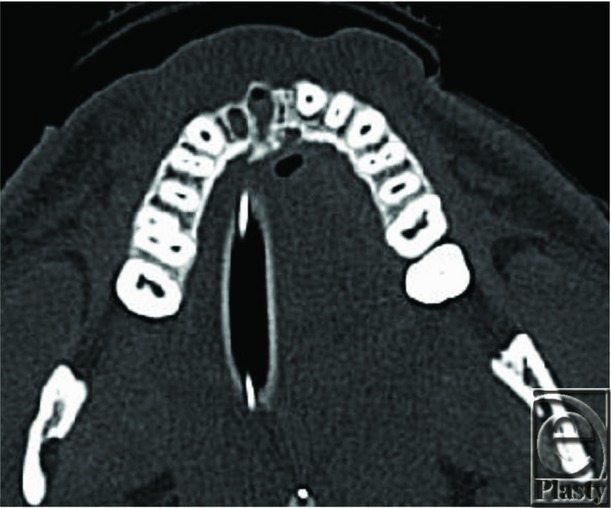
Admission computed tomographic scan illustrating avulsion of teeth and fracture of alveolar ridge.
